# Molecular determinants for regulation of G3BP1/2 phase separation by the SARS-CoV-2 nucleocapsid protein

**DOI:** 10.1038/s41421-021-00306-w

**Published:** 2021-08-17

**Authors:** Wenjie Huang, Xiaohui Ju, Min Tian, Xiaoyu Li, Yanying Yu, Qingxiang Sun, Qiang Ding, Da Jia

**Affiliations:** 1 Key Laboratory of Birth Defects and Related Diseases of Women and Children, Department of Paediatrics, West China Second University Hospital, State Key Laboratory of Biotherapy and Collaborative Innovation Center of Biotherapy, Sichuan University, Chengdu, China; 2 School of Medicine, Tsinghua University, Beijing, China

**Keywords:** Cell signalling, Organelles

Dear Editor,

The organization and regulation of membraneless compartments in cells remain one of the fundamental questions in biology^1^. How cells harness condensates for their well-being and how pathogens hijack or counteract condensates for their proliferation are elusive. Stress granules (SGs) are dynamic large cytoplasmic phase-separated mRNA-protein condensates in response to various stresses. The assembly of SGs can be positively or negatively regulated by many endogenous or exogenous factors^2,3^. G3BP1/2 are the core components of stress granules, which undergoes RNA-dependent liquid–liquid phase separation (LLPS)^2,3^. G3BP1/2 interact with a large number of proteins, including Caprin-1 and TIA-1, and both of them promote G3BP1/2-mediated LLPS ^2,3^. Very recently, G3BP1/2 is shown to interact with the nucleocapsid (N) protein of SARS-CoV-2^4–7^. In contrast to Caprin-1 and TIA-1, N protein inhibits G3BP1/2-mediated LLPS^4,6^. Although the regulation of G3BP1/2-mediated LLPS by N protein is likely to be critical for the SARS-CoV-2 production^6^, the nature of the G3BP1/2-N interaction and the mechanisms underlying the different regulation of G3BP1/2 remain unclear.

SARS-CoV-2, like SARS-CoV and MERS-CoV, is a type β coronavirus with single-stranded RNA. Among ~29 proteins encoded by the SARS-CoV-2 genome, N protein is mainly responsible for RNA packaging and facilitating viral RNA synthesis through recruiting host factors. N protein encompasses of two conserved domains: the N-terminal RNA recognition motif (RRM) and the C-terminal dimerization domain (Dimerization) (Fig. 1a). The two domains are flanked by three intrinsically disordered regions (IDRs): the N-terminal IDR (aa1–48), the central IDR enriched with serine and arginine (SR-IDR), and C-terminal IDR (Fig. 1a). N protein also undergoes LLPS in the presence of RNA, with RRM, Dimerization domain, and the central IDR all play an important role in the process^5,8^.

**Fig. 1:**
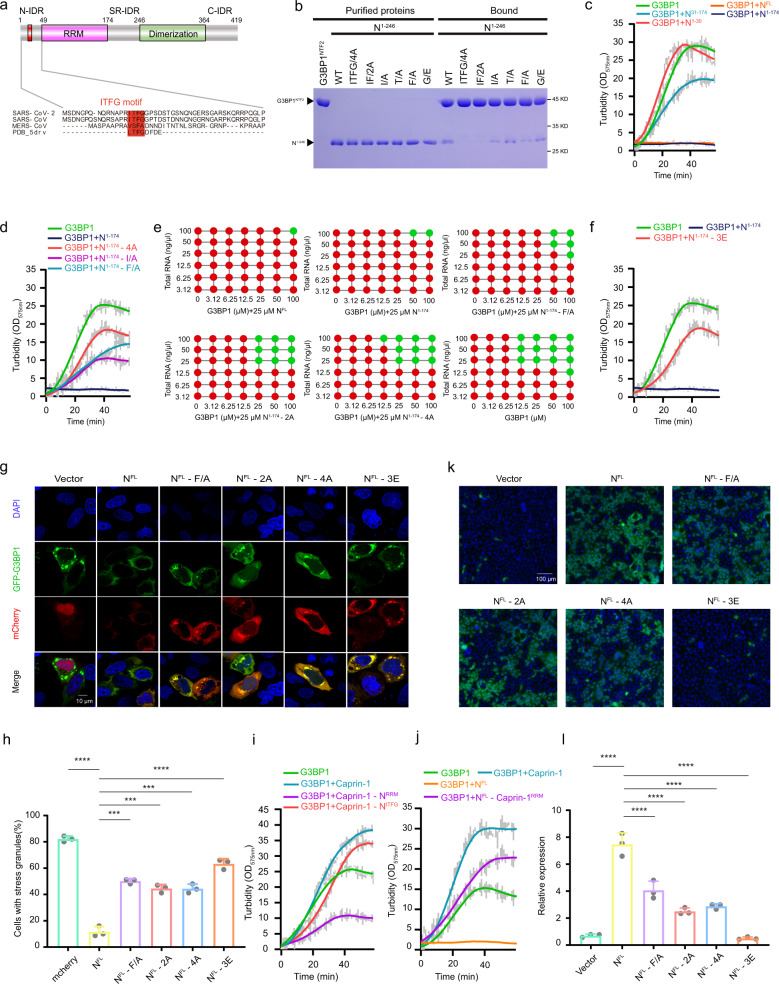
SARS-CoV-2 N protein utilizes two components, the ITFG motif and RRM, to regulate G3BP1 phase separation. **a** Schematic diagram of SARS-CoV-2 N protein highlights an ITFG motif located in the N-IDR. IDR intrinsically disordered region. **b** GST–G3BP1^NTF2^ pull-down of purified SARS-CoV-2 N^1–246^ and ITFG mutants. **c** SARS-CoV-2 N^1–174^, but not N^1–30^ or N^30–174^, is sufficient to inhibit phase separation of G3BP1 in the presence of RNA. Equimolar N fragment was mixed with MBP-G3BP1. TEV was added in solution to cleave the MBP fusion, and OD_575nm_ was recorded over time. Data represent mean±s.d. from 3 independent experiments. **d** Mutations in the ITFG motif of N protein impaired its ability to inhibit G3BP1-mediated phase separation. Experiments were performed similar to (**c**). **e** Summary of the phase separation behaviors of purified recombinant G3BP1 and RNA, in the absence of N protein or the presence of various N mutants. Red: no droplet; green: droplet. **f** Mutation of three basic amino acids (R92E/R107E/R149E, 3E) in the RRM domain of SARS-CoV-2 N protein significantly impaired the inhibitory effect of N protein on G3BP1 phase separation. **g**, **h** Overexpression of SARS-CoV-2 N protein inhibited the formation of SGs in cells, and mutation of the ITFG motif or RRM significantly abrogated the inhibition. Data shown in (**h**) represent mean ± s.d. from three independent experiments. Results were evaluated by one-way ANOVA test (**P < 0.01; ***P < 0.001; ****P < 0.0001; NS, not significant). Scale bar: 10 μm. **i** Turbidity of G3BP1 with Caprin-1 or its mutants over time in the presence of RNA. Data represent mean ± s.d. from three independent experiments. **j** Turbidity of G3BP1 with Caprin-1 or N protein over time in the presence of RNA. Data represent mean ± s.d. from three independent experiments. **k**, **l** Caco-2 cells expressing N wild-type or mutants were infected with SARS-CoV-2 GFP/ΔN trVLP, and cell culture supernatant was collected to infect Caco-2-N wild-type cells. GFP signal was observed using microscopy, and cellular RNA was extracted for RT-qPCR analysis to determine viral subgenomic RNA levels. Data represent mean ± s.d. from three independent experiments. *P < 0.05; **P < 0.01; ***P < 0.001; ****P < 0.0001. Significance assessed by one-way ANOVA. Scale bar: 100 μm.

To determine the regions of G3BP1/2 and N protein that are responsible for their interaction, we purified a serial of G3BP1 and N truncation proteins and performed GST pull-down assays (Supplementary Fig. S1a, b). These results indicated that a fragment from the N-IDR of N protein (N^11–24^) and the NTF2 domain of G3BP1 (G3BP1^NTF2^) are the minimal fragments required for the interaction (Supplementary Fig. S1a, b). Interestingly, the N-IDR of N protein harbors a sequence (we termed as the ITFG motif) similar to that of a peptide known to interact with G3BP1^NTF2^ (Fig. 1a). This motif is absolutely conserved in the N protein of SARS-CoV, and only partially conserved in MERS-CoV. To evaluate the contribution of this motif for the N-G3BP1 interaction, we introduced single, double, and quadruple mutations in the motif. Both GST pull-down and ITC assays demonstrated that all 4 residues within the ITFG motif contribute to the binding, with I and F making the greatest contribution (Fig. 1b and Supplementary Fig. S1c). N^11–24^ bound to G3BP1^NTF2^ with an affinity of ~10 μM, and the affinity was reduced by 6~7-fold for both the I/A and F/A mutations (Supplementary Fig. S1c). And no-binding was detected for the quadruple mutation (4A) (Fig. 1b and Supplementary Fig. S1c). Similarly, we identified three hydrophobic amino acids (F15, F33, F124) in G3BP1^NTF2^ critical for binding to N protein^9^ (Supplementary Fig. S1d, e). Consistent with our sequence alignment, we found that N protein of SARS-CoV, but not that of MERS-CoV, strongly interacted with G3BP1 (Fig. 1a and Supplementary Fig. S1f). Furthermore, consistent with the high similarity between the NTF2 domains of G3BP1 and G3BP2, N protein of SARS-CoV-2 interacts with G3BP2 in a similar manner (Supplementary Fig. S1g).

We next determined which region of N protein is responsible for G3BP1-mediated LLPS. Under our experimental condition, N^1–174^ pronouncedly inhibited G3BP1-mediated LLPS in the presence of RNA, to an extent similar to N^FL^ (Fig.1c). Interestingly, N^1–174^ did not form phase-separated droplets in vitro, unlike N^FL^ (Supplementary Fig. S2a). Thus, N protein regulates G3BP1-mediated LLPS independent of its own LLPS property. Further truncation of N protein indicated that both the N-IDR and RRM were required for inhibiting G3BP1-mediated LLPS (Fig. 1c). To understand the contribution of the ITFG motif for the inhibitory effect of N protein, we tested how the corresponding mutations affected G3BP1-mediated LLPS (Fig. 1d, e). In contrast to N^1–174^ WT, single (I/A, F/A) or quadruple mutations (4A) greatly reduced the inhibitory effect of N protein (Fig. 1d, e). Conversely, N^1–174^ WT failed to inhibit G3BP1-3A-mediated LLPS (Supplementary Fig. S2b). Furthermore, a triple- mutation that affects the RRM residues critical for RNA binding (R92E/R107E/R149E, referred to as 3E) also weakened the inhibitory effect of N protein on G3BP1-mediated LLPS (Fig. 1f and Supplementary Fig. S2c). Remarkably, in contrast with N^FL^ WT, both the 4A and 3E mutants failed to inhibit G3BP1-mediated LLPS, further confirming that the LLPS property of N protein is independent of its function in inhibiting G3BP1-mediated LLPS (Supplementary Fig. S2d). In line with these in vitro studies, we found that both the ITFG motif and RRM of N protein are critical for dissembly SGs in vivo (Fig. 1g, h). Taken together, our data demonstrate that N protein regulates G3BP1/2-mediated LLPS via both the ITFG motif and RRM.

We next sought to determine why N protein and other stress granule proteins differentially regulated G3BP1/2-mediated LLPS. Caprin-1 is an RNA-binding protein widely found in vertebrates, and mediates the transport and translation of mRNA^10^. Caprin-1 and N protein share a similar domain structure, including a G3BP1/2-binding motif, RRM, and a dimerization domain (Supplementary Fig. S2e). To test why Caprin-1 promotes G3BP1/2-mediated LLPS whereas N protein inhibits, we generated chimera constructs in which the G3BP1/2-binding motif and RRM were replaced by that of N protein, respectively (Supplementary Fig. S2e). Replacing the G3BP1-binding motif of Caprin-1 with the ITFG motif of N protein (Caprin-1-N^ITFG^) did not drastically alter the regulation of G3BP1 by Caprin-1 (Fig. 1i and Supplementary Fig. S2e). In contrast, substitution of RRM of Caprin-1 with that of N protein (Caprin-1-N^RRM^) reversed the activity of Caprin-1, and made it as an inhibitor in regulating G3BP1-mediated LLPS (Fig. 1i and Supplementary Fig. S2f). Conversely, substitution of the RRM of N protein with that of Caprin-1 (N^FL^-Caprin-1^RRM^) switched N protein from an inhibitor to an activator of G3BP1-mediated LLPS (Fig. 1j). These data indicate that the RNA-binding property could determine how a modulator protein differentially regulates phase separation of SGs, or ribonucleoprotein granules in general.

N protein and G3BP1 have a similar RNA-binding property and both prefer to binding mRNAs in their 3′UTRs^4^. Other studies have shown that N protein has a greater preference for RNA non-coding regions enrich with UCUAA^11^; in contrast, Caprin-1 binds to the coding regions of mRNA^12,13^. The similar RNA-binding property could explain the anti-cooperativity between N and G3BP1/2 in RNA-mediated LLPS. On the other hand, the direct interaction between G3BP1/2 and Caprin-1, coupled with the abilities of targeting different regions of mRNA, could dramatically increase the valence in the system, thus explaining the cooperativity of G3BP1/2 and Caprin-1 in SG assembly. If our hypothesis is correct, replacing of the RRM of N protein with a similar RNA-binding domain should retain the inhibitory effect of N protein. Indeed, a chimera construct which consists of the N-IDR region of N protein and the RRM of HuR^14^ significantly inhibited G3BP1-mediated LLPS (Supplementary Fig. S2e, g).

We have previously developed a biosafety level-2 cell culture system for production of transcription and replication-competent SARS-CoV-2 virus-like-particles (trVLP)^15^. This trVLP expresses a reporter gene (GFP) replacing viral N gene (SARS-CoV-2 GFP/ΔN trVLP). The complete viral life cycle can be achieved and exclusively confined in the cells ectopically expressing SARS-CoV-2 N protein. Using this system, we found that deletion of N-IDR completely abrogated SARS-CoV-2 production^6^. To precisely define the roles of the N-G3BP1/2 interaction in SARS-CoV-2 production, Caco-2 cells were lentivirally transduced with N with mutations in the ITFG motif. The transduced cells were subsequently infected with SARS-CoV-2 GFP/ΔN trVLP at a multiplicity of infection of 0.05. After 24 h, cell culture supernatants were collected to infect the naive Caco-2-N wild-type cells. After another 24 h, GFP expression, which is proxy of the trVLP production from the Caco-2 cells expressing mutant N proteins, was analyzed by microscopy (Fig. 1k) and flow cytometry (Supplementary Fig. S3a). In addition, RT-qPCR was performed to analyze subgenomic RNA level (Fig. 1l). Our results showed that N protein mutants (F/A, 2A, 4A) that could not interact with G3BP1/2 are impaired in mediating SARS-CoV-2 trVLP production, suggesting the importance of interaction between N and G3BP1/2 in SARS-CoV-2 viral life cycle (Fig. 1k, l and Supplementary Fig. S3a). We also tested the importance of RRM for SARS-CoV-2 production. Unfortunately, 3E mutant are not stable in Caco-2 cells due to unknown reasons (Supplementary Fig. S3b, c). In contrast, all the ITFG mutants were normally expressed in both 293T and Caco-2 cells (Supplementary Fig. S3b, c).

In summary, we present a “two-component” mechanism whereby the N protein and other factors differentially regulate G3BP1-mediated LLPS (Supplementary Fig. S4). We show that both components of the SARS-CoV-2 N protein, the ITFG motif and the RNA-binding domain, are required for the inhibition of G3BP1-mediated LLPS. The ITFG motif of the N protein is sufficient to interact with G3BP1/2, whereas the RNA-binding property determines whether a protein positively or negatively regulate SG formation. Based on their selectivity to RNA binding, each regulatory factor may stabilize or destabilize the dynamic stability of the protein–RNA interaction network formed by G3BP1/2 (Supplementary Fig. S4). For SARS-CoV-2, the N protein is able to inhibit the formation of SG in host cells, thereby inhibiting the innate immune pathways and cell apoptosis, and ultimately promoting its own proliferation^16,17^. Altogether, our study provides a simplified model that explains different regulation of SG assembly, and may represent a general principle in controlling the phase separation properties of ribonucleoprotein granules.

## Supplementary information

Supplementary figures
