# Supplementary material for: Molecular determinants for regulation of G3BP1/2 phase separation by the SARS-CoV-2 nucleocapsid protein
Source: Cell Discov. 2021 Aug 17;7:69. doi: 10.1038/s41421-021-00306-w (PMC8368218; doi:10.1038/s41421-021-00306-w)
Supplement: Supplementary file 1 — Supplementary figures [file 41421_2021_306_MOESM1_ESM.pdf]

Molecular determinants for regulation of G3BP1/2 phase separation by the SARS-CoV-2 Nucleocapsid protein

Wenjie Huang[1,†], Xiaohui Ju[2,†], Min Tian[1†], Xiaoyu Li[1†], Yanying Yu[2], Qingxiang Sun[1], Qiang Ding[2,*], Da Jia[1,*]

[1]Key Laboratory of Birth Defects and Related Diseases of Women and Children, Department of Paediatrics, West China Second University Hospital, State Key Laboratory of Biotherapy and Collaborative Innovation Center of Biotherapy, Sichuan University, Chengdu 610041, China

[2] School of Medicine, Tsinghua University, Beijing, China

[†]Equal contribution.

*To whom correspondence should be addressed.

Qiang Ding, E-mail: qding@tsinghua.edu.cn;

Da Jia, E-mail: Jiada@scu.edu.cn.

Methods

Molecular cloning, protein expression and purification

The SARS-CoV-2 N protein-coding fragments were synthesized, and cloned into the pET28a vector, encoding a N-terminal His*6 tag. The human G3BP1 gene was PCR-amplified from a human cDNA library, and cloned into a modified pGEX-4T1 or PMAL vector, yielding an N-terminal cleavable GST or MBP fusion. Proteins were expressed purified as a previous report [1]. Most experiments were performed with His-tagged N-protein and GST-tagged G3BP1 except for the *in vitro* LLPS assay, which were performed with MBP-tagged G3BP1.

GST Pull-down

GST pull-down assays were carried out similar to a previous study [1]. The proteins (20 µg of GST or GST-tagged protein, and 300 µg of bait proteins) were mixed with glutathione-Sepharose beads (20 µl) in 0.5 mL of PD buffer (50 mM Tris pH7.5, 300 mM NaCl, 0.05% Triton-X100). After binding at 4°C for 1 hr, the beads were washed three times with 1 ml of PD buffer. The samples were separated on 15% SDS-PAGE, and visualized by Coomassie staining or immunoblotting.

Isothermal Titration Calorimetry (ITC)

ITC experiments were performed on a Microcal iTC200 instrument, as previously described [1]. Prior to the experiment, proteins and peptides were dialyzed to the ITC buffer (100mM Tris pH7.5, 300 mM NaCl, 5% v/vGlycerol). ITFG peptides (1.0 mM)

were titrated into G3BP1^{NTF2} domain (50 μM) at 25 °C. Each experiment was performed at least three times. Data were analyzed using origin 7.0 .

LLPS assay

In vitro LLPS experiments were performed at room temperature, similar to previous studies [2, 3]. N protein was incubated with MBP-tagged G3BP1 in solution (50 mM Tris pH 7.5, 150 mM NaCl, 1 mM DTT, 1 mM EDTA, 10% v/v Ficoll, 25 ng/ul desalted torula yeast RNA extract) for 10 minutes, before the addition of TEV protease, which cleaves the MBP tag. Turbidity experiments were performed in a 96-well clear plate (Costar), and absorbance at 575 nm (OD_{575nm}) was monitored over time at 1 min time intervals for up to 1 hour with mixing. The turbidity of “no TEV control” was recorded and subtracted. Each experiment was conducted independently at least three times.

The microscopy imaging experiment was similarly performed [2, 3]. Proteins were mixed and transferred to a sandwiched chamber, and droplet formation was evaluated using a Leica DMi8 microscope with a 30x objective. Imaged were captured within 15 min after LLPS induction.

Quantification of stress granules in cells

HeLa cells (ATCC) were grown in DMEM medium (Gibco) with 10% fetal bovine serum (BI) at 37℃with 5% CO_2 . The cells were co-transfected with GFP-G3BP1

FL and mCherry-N wild-type or mutants. Twenty-four hours after the transfection, the cells were washed with PBS and fixed with 4% formaldehyde at room temperature. Confocal images were acquired by Olympus FV-1000 confocal microscope. Cells with at least 3 granules were counted as stress granule-positive, similar to previous studies [2, 3].

Lentiviral plasmid construction

pLVX-IRES-mCherry was digested with XhoI and XbaI to prepare backbone. N gene was amplified by PCR with primer THU-2197 (5'-TCTATTTCCGGTGAATTCCTCGAGAT GTCTGATAATGGACCCCAAAATCAG-3') and primer THU-2198 (5'-GGCGGGATCCGCGGCCGCTCTAGATTACTTATCGTCGTCATCCTTGTAATCT C-3'). The ligation was completed by ClonExpress MultiS One Step Cloning Kit (C113, Vazyme). DNA sequences were verified by Sanger DNA sequencing.

Lentivirus package and transduction

Vesicular stomatitis virus G protein (VSV-G) pseudotyped lentiviruses were produced by transient cotransfection of packaging plasmids pMD2G (catalog no. 12259; Addgene), psPAX2 (catalog number 12260; Addgene) and the transfer vector pLVX-IRES-zsGreen by VigoFect DNA transfection reagent (Vigorous) into HEK293T cells. Culture medium was changed 12 h post transfection. Supernatants were collected at 36, 60 and 84 h after transfection, pooled, passed through a 0.45-μm

filter, aliquoted, and frozen at -80°C refrigerator.1×105 Caco-2 cells were seeded into one well of 24-well plate one day before, cells are infected with lentivirus added with 10mg/ml polybrene. 96 hours post transduction, western blot was performed to analyze N expression.

SARS-CoV-2 GFP/ΔN trVLP production and infection

SARS-CoV-2 GFP/ΔN trVLP is prepared as previously described [4]. Caco-2 cells expressing wild-type or mutant N were seeded into a 24-well cell plate, after 16 hours, the cells were infected with SARS-CoV-2 GFP/ΔN trVLP P5 at a multiplicity of infection (MOI) of 0.05.

RNA isolation and RT-qPCR

Total cellular RNA was extracted with Trizol reagent (Thermo, 15596018). To analyze viral RNA level in cells, quantitative real-time PCR was performed. In brief, 1μg total RNA was reverse transcribed using ReverTra Ace qPCR RT Kit (TOYOBO, FSQ-101) to produce cDNA with random primers. Reactions of qPCR were performed using the 2×RealStar Green Power Mixture (Genstar, A311) according to the instruction. The qPCR primers for viral RNA were as follows: THU-2816 (5'-CGATCTCTTGTAGATCTGTTCTC-3') and THU-2818 (5'- TCAGGGTCAGCTT GCCGTAG-3'). The sequences of the qPCR primers for GAPDH were described previously [5]. Relative expression levels of the target genes were calculated using the comparative cycle threshold (CT) method. All data were normalized relative to the

housekeeping gene GAPDH.

Flow cytometry analysis

Cells were digested in 0.25% trypsin and then washed once with cold PBS. Cells were fixed in 4% PFA for 30 minutes at room temperature. Fixed cells were resuspended in PBS and analyzed by LSRFortessa SORP (BD Biosciences) and FlowJo software.

Statistical analysis

All viral and cellular experiments were performed at least three times. Statistical analyses were performed using one-way ANOVA, Tukey's multiple comparisons test incorporated in Prism 8 (GraphPad Software). ****P < 0.0001,***P < 0.0005, **P < 0.01, *P < 0.05, ns:not significant.

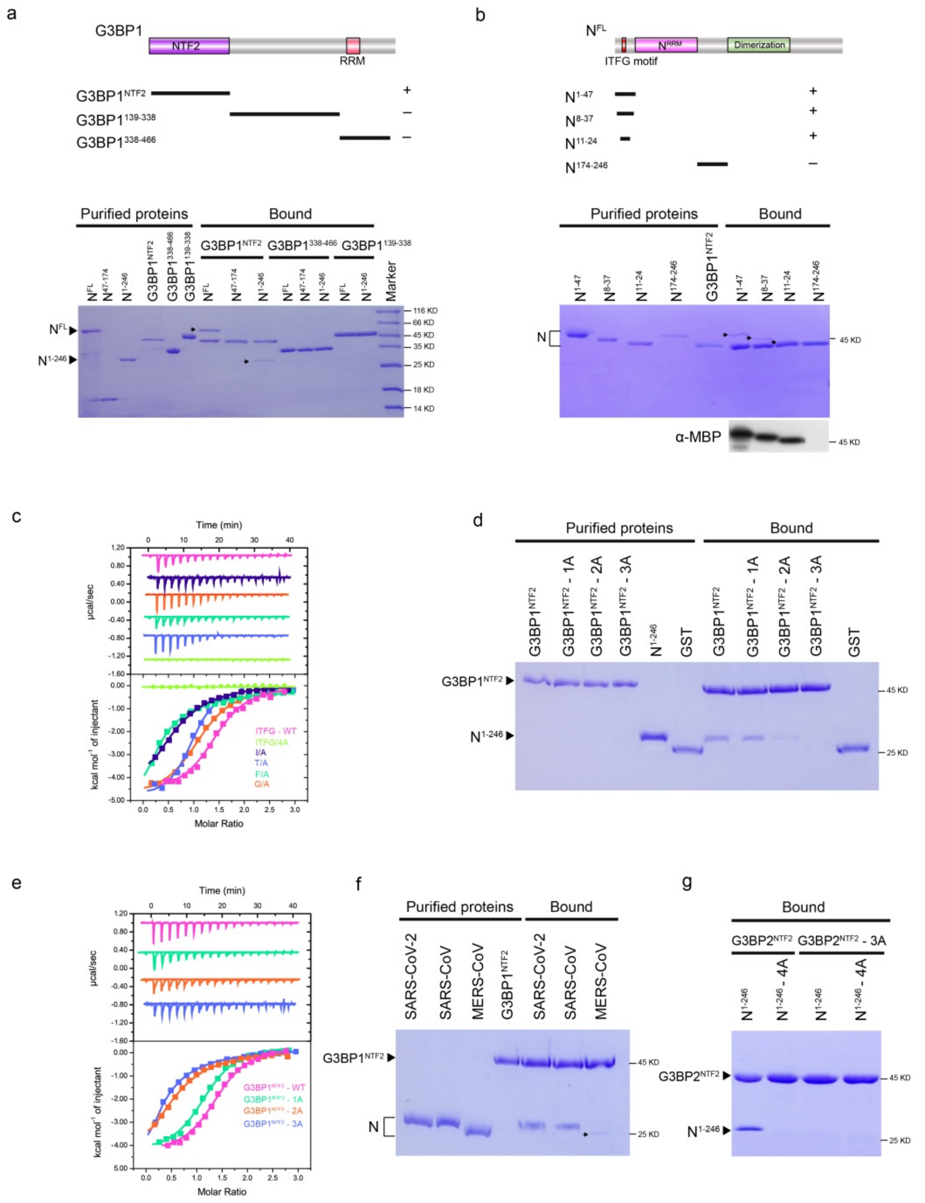

Supplementary Fig.S1 SARS-CoV-2 N protein directly interact with the NTF2 domain of G3BP1/2 through an ITFG motif.

a GST–G3BP1 fragments pull-down of full-length N protein or its fragments. The bound proteins were subjected to Coomassie stained SDS-PAGE.

b GST–G3BP1^{NTF2} pull-down of various fragments of MBP-tagged N protein. The bound proteins were subjected to Coomassie-stained SDS-PAGE or immunoblotting using anti-MBP antibody.

c Isothermal titration calorimetry of an ITFG-bearing peptide (NAPRITFGGPSDST) or mutants titrated into G3BP1^{NTF2} in a buffer containing 100mM Tris pH 7.5, 300 mM NaCl, 5% v/v Glycerol at 25 °C. Top and bottom panels show raw and integrated heat from injections, respectively. The curves of different colors in the bottom panel represent a fit of the integrated data to a single-site binding model.

d GST–G3BP1^{NTF2} or its mutants 1A (F15A), 2A (F15A/F33A), 3A (F15A/F33A/F124A) pull-down of purified N$^{1-246}$.

e Isothermal titration calorimetry of ITFG peptide(NAPRITFGGPSDST) titrated into G3BP1^{NTF2} or mutants in a buffer containing 100mM Tris pH 7.5, 300 mM NaCl, 5% v/v Glycerol at 25 °C.

f GST–G3BP1^{NTF2} pull-down of purified SARS-CoV-2 N$^{1-246}$, SARS-CoV N$^{1-246}$ and MERS-CoV N$^{1-235}$.

g GST–G3BP2^{NTF2} and its 3A (F15A/F33A/F124A) mutant pull-down of purified SARS-CoV-2 N$^{1-246}$ or N$^{1-246}$ – 4A.

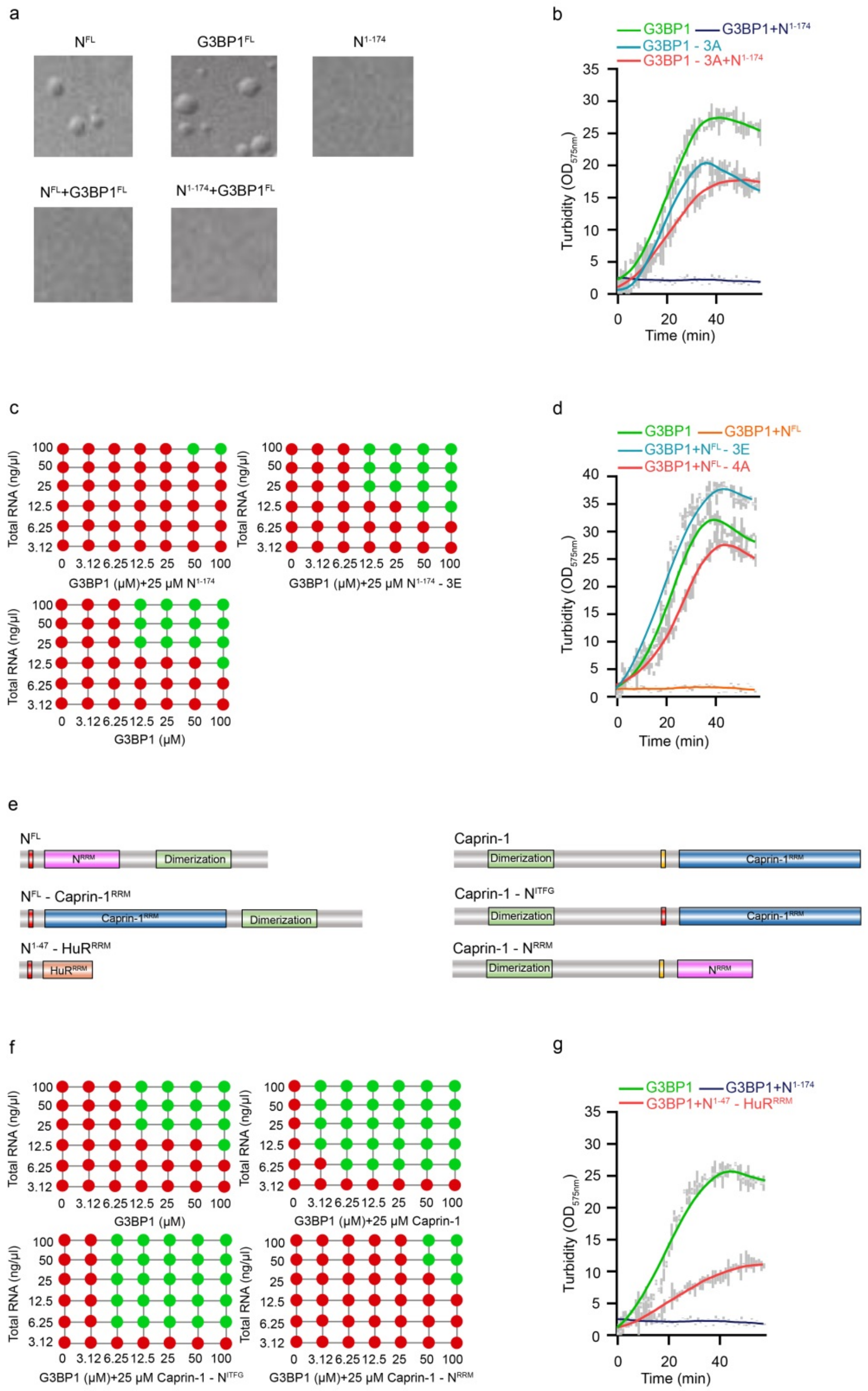

Supplementary Fig.S2 RNA-binding property determines whether a regulator protein positively or negatively G3BP1-mediated LLPS.

a DIC images of phase separation of N protein, G3BP1, and their mixture in the

presence of a 25 ng/ul total RNA and 150 mM NaCl.

b Turbidity of G3BP1 or its 3A (F15A/F33A/F124A) mutant together with $N^{1\text{-}174}$ in the presence of RNA. Data represent mean ± s.d. from 3 independent experiments.

c Summary of the phase separation behaviors of purified recombinant G3BP1 and RNA, in the absence of N protein or in the presence of N wild-type or 3E mutant. Red: no droplet; green: droplet.

d Turbidity of G3BP1 together with N^{FL} WT , N^{FL} – 4A(I15A/T16A/F17A/G18A) or N^{FL} – 3E(R92E/R107E/R149E) mutants in the presence of RNA. Data represent mean ± s.d. from 3 independent experiments.

e Schematic diagram of various constructs used in this study. Red and yellow bars represent the ITFG motif of N protein and the G3BP1-binding region of Caprin-1, respectively.

f Summary of the phase separation behaviors of purified recombinant G3BP1 and RNA, in the absence of Caprin-1 or in the presence of Caprin-1 wild-type or mutants. Red: no droplet; green: droplet.

g Turbidity of G3BP1 together with $N^{1\text{-}174}$ or $N^{1\text{-}174}$-HuR^{RRM} in the presence of RNA. Data represent mean ± s.d. from 3 independent experiments. $N^{1\text{-}47}$-HuR^{RRM} encompasses the N-IDR region of the N protein and the RRM (100-174aa) of HuR.

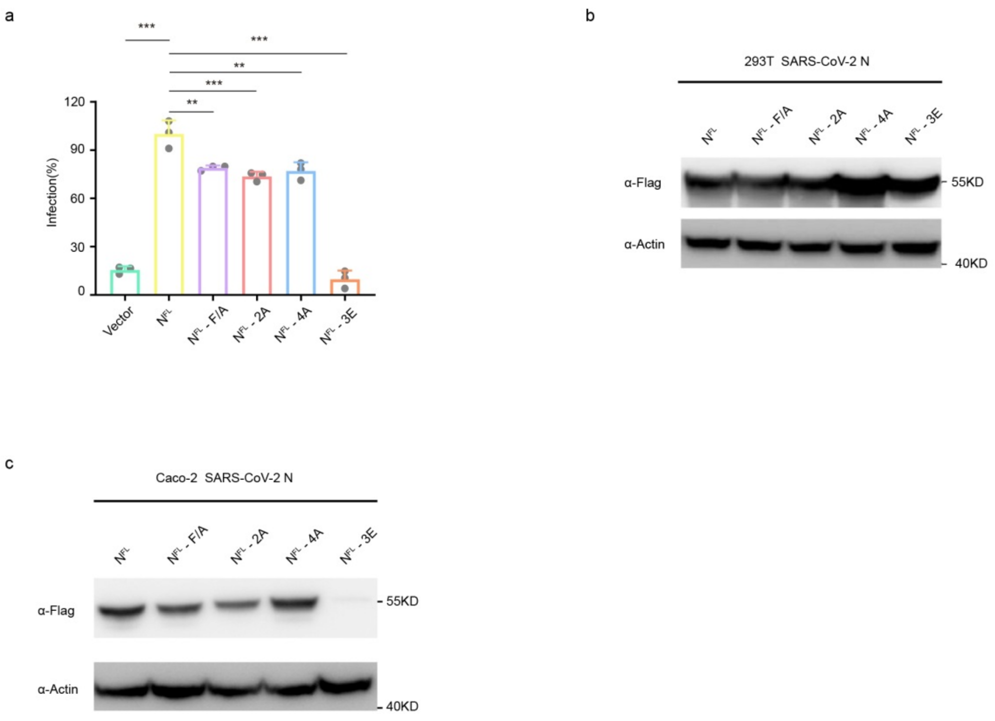

Supplementary Fig.S3 The interaction between N and G3BP1 is important for SARS-CoV-2 life cycle.

a The cell culture medium was collected from SARS-CoV-2 GFP/ΔN trVLP infected Caco-2 cells expressing N wild-type or mutants to infect the naive Caco-2-N wild-type cells. After 24 hours, GFP expression was quantified by flow cytometry. Error bars represent the standard deviations from one of three independent experiments performed in triplicate. *, $P < 0.05$; **, $P < 0.01$; ***, $P < 0.001$. Significance assessed by one-way ANOVA.

b Immunoblotting was used to detect the expression of N proteins in 293T cells expressing N wild-type or mutants.

c Immunoblotting was used to detect the expression of N proteins in Caco-2 cells expressing N wild-type or mutants.

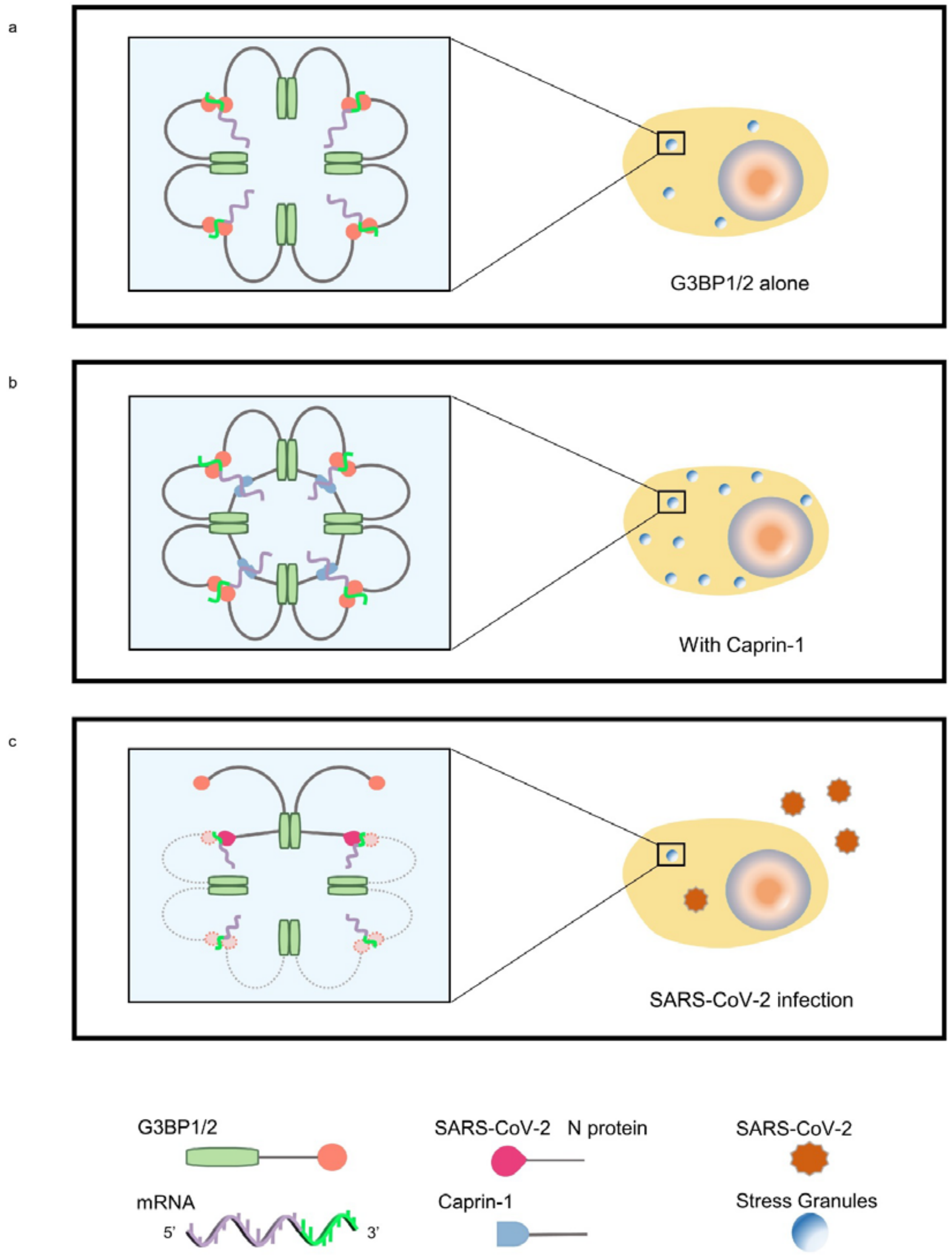

Supplementary Fig.S4 A model explaining how SARS-CoV-2 N protein and Caprin-1 differentially regulate phase separation of G3BP1/2

a Under normal condition, G3BP1/2 dimerize via the NTF2 domain and form a liquid-liquid phase separation condensate through multivalent interaction with mRNA.

b Caprin-1 directly binds to the NTF2 domain of G3BP1/2. G3BP1/2 and Caprin-1 bind different regions of mRNA, thereby stabilizing and enhancing the interaction network formed by G3BP1/2 and mRNA.

c SARS-CoV-2 N protein, via its ITFG motif, directly binds to the NTF2 domain of G3BP1/2. As N and G3BP1/2 likely bind to similar mRNA regions, N protein destabilizes the G3BP1/2-RNA network. Inhibition of SGs by N protein could promote SARS-CoV2 production via manipulating host antiviral response.

REFERENCES

1. Huang, W., et al., *Structural and functional studies of TBC1D23 C-terminal domain provide a link between endosomal trafficking and PCH.* Proc Natl Acad Sci U S A, 2019. **116**(45): p. 22598-22608.
2. Guillen-Boixet, J., et al., *RNA-Induced Conformational Switching and Clustering of G3BP Drive Stress Granule Assembly by Condensation.* Cell, 2020. **181**(2): p. 346-361 e17.
3. Yang, P., et al., *G3BP1 Is a Tunable Switch that Triggers Phase Separation to Assemble Stress Granules.* Cell, 2020. **181**(2): p. 325-345 e28.
4. Ju, X., et al., *A novel cell culture system modeling the SARS-CoV-2 life cycle.* PLoS Pathog, 2021. **17**(3): p. e1009439.
5. Ju, X., et al., *Identification of functional cis-acting RNA elements in the hepatitis E virus genome required for viral replication.* PLoS Pathog, 2020. **16**(5): p. e1008488.
